# Link Prediction in Complex Networks: A Mutual Information Perspective

**DOI:** 10.1371/journal.pone.0107056

**Published:** 2014-09-10

**Authors:** Fei Tan, Yongxiang Xia, Boyao Zhu

**Affiliations:** Department of Information Science and Electronic Engineering, Zhejiang University, Hangzhou, Zhejiang, China; University of Maryland, College Park, United States of America

## Abstract

Topological properties of networks are widely applied to study the link-prediction problem recently. Common Neighbors, for example, is a natural yet efficient framework. Many variants of Common Neighbors have been thus proposed to further boost the discriminative resolution of candidate links. In this paper, we reexamine the role of network topology in predicting missing links from the perspective of information theory, and present a practical approach based on the mutual information of network structures. It not only can improve the prediction accuracy substantially, but also experiences reasonable computing complexity.

## Introduction

Link prediction attempts to estimate the likelihood of the existence of links between nodes based on the available network information, such as the observed links and nodes' attributes [1], [2]. On the one hand, the link-prediction problem is a long-standing practical scientific issue. It can find broad applications in both identifying missing and spurious links and predicting the candidate links that are expected to appear with the evolution of networks [1], [3], [4]. In biological networks (such as protein-protein interaction networks [5] and metabolic networks [6]), for example, the discovery of interactions is usually costly. Therefore, accurate prediction is more reasonable compared with blindly checking all latent interaction links [3], [4]. In addition, the detection of inactive or anomalous connections in online social networks may improve the performance of link-based ranking algorithms [7]. Furthermore, in online social networks, very promising candidate links (non-connected node pairs) can be recommended to the relevant users as potential friendships [8], [9]. It can help them to find new friends and thus enhance their loyalties to the web sites. In ref. [9], the authors even proposed the potential theory to facilitate the missing link prediction of directed networks. The hypothesis can find broad applications in friendship recommendation of large-scale directed social networks, such as Twitter, Weibo and so on. On the other hand, theoretically, link prediction can provide a useful methodology for the modeling of networks [10]. The evolving mechanisms of networks have been widely studied. Many evolving models have been proposed to capture the evolving process of real-world networks [11]–[14]. However, it is very hard to quantify the degree to which the proposed evolving models govern real networks. Actually, each evolving model can be viewed as the corresponding predictor, we can thus apply evaluating metrics on prediction accuracy to measure the performance of different models.

Therefore, link prediction has attracted much attention from various scientific communities. Within computer society, for example, scientists have employed Markov chains [15], [16] and machine learning techniques [17]–[21] to extract features of networks. These methods, however, depend on the attributes of nodes for particular networks such as social and textual features. Obviously, the attributes of nodes are generally hidden, and it is thus difficult for people to obtain them [2].

Over the last 15 years, network science has been developed as a novel framework for understanding structures of many real-world networked systems. Recently, a wealth of algorithms based on structural information have been proposed [2], [4], [22]–[28]. Among various node-neighbor-based indices, Common Neighbors (CN) is undoubtedly the precursor with low computing complexity. It has also been revealed that CN achieves high prediction accuracy compared with other classical prediction indices [25]. CN, however, only emphasizes the number of common neighbors but ignores the difference in their contributions. In this case, several variants of CN to correct such a defect were put forwarded. Consider, for example, Adamic-Adar [24] and Resource Allocation [25], in which low-degree common neighbors are advocated by assigning more weight to them. In addition, based on the Bayesian theory, a Local Nave Bayes model [27] was presented to differentiate the roles of neighboring nodes. Furthermore, node centrality (including degree, closeness and betweenness) was applied to make neighbors more distinguishable. Besides such CN-based indices, the evolving patterns and organizing principles of networks can also provide useful insights for coping with the link-prediction problem. The well-known mechanism of preferential attachment [11], for instance, has been viewed as a prediction measure [25], [29]. For networks exhibiting hierarchical structure, Hierarchical Random Graph can be employed to predict missing links accordingly [4]. Recently, communities have been reinvented as groups of links rather than nodes [30]. Motivated by the shift in perspective of communities, Cannistraci et al. developed the local-community-paradigm to enhance the performance of classical prediction techniques [28].

All the aforementioned methods aim to quantify the existence likelihood of candidate links. In information theory, the likelihood can be measured by the self-information. In this article, we thus try to give a more theoretical analysis of the link-prediction problem from the perspective of information theory. Then a general prediction approach based on mutual information is presented accordingly. Our framework outperforms other prediction methods greatly.

## Results

### A Mutual Information Approach to Link Prediction

We here introduce the definitions of the self-information and of the mutual information, respectively.

**Definition 1** Considering a random variable 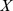 associated with outcome 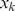 with probability 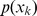, its *self-information* 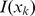 can be denoted as [31]

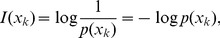
(1)where the base of the logarithm is specified as 2, thus the unit of self-information is bit. This is applicable for the following if not otherwise specified. The self-information indicates the uncertainty of the outcome 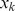. Obviously, the higher the self-information is, the less likely the outcome 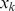 occurs.

**Definition 2** Consider two random variables 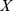 and 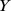 with a joint probability mass function 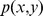 and marginal probability mass functions 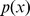 and 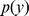. The *mutual information* 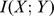 can be denoted as follows [32]: 
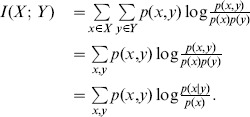
(2)


Hence, the mutual information 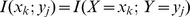 can be obtained as 
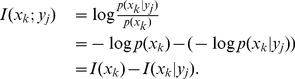
(3)


The mutual information is the reduction in uncertainty due to another variable. Thus, it is a measure of the dependence between two variables. It is equal to zero if and only if two variables are independent.

Now consider the link-prediction problem. Our idea is to use the local structural information to facilitate the prediction. To do that, we denote the set of node 
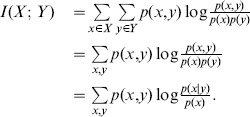
's neighboring nodes by 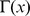. For node pair 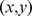, the set of their common neighbors is denoted as 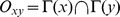.

Given a disconnected node pair 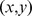, if the set of their common neighbors 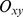 is available, the likelihood score of node pair 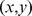 is defined as 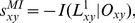
(4)where 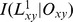 is the conditional self-information of the existence of a link between node pair 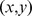 when their common neighbors are known. According to the property of the self-information, the smaller 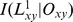 is, the higher the likelihood of existence of links is. Thus, we define the score as the negation of 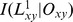. According to the definition of mutual information, 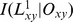 can thus be derived as 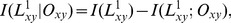
(5)where 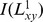 is the self-information of that node pair 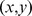 is connected. 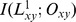 is the mutual information between the event that node pair 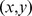 has one link between them and the event that the node pair's common neighbors are known. Note that 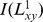 is calculated by the prior probability of that node 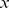 and node 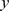 are connected. In our method, without knowing the common neighbors of node pair 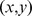, we could use 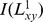 to estimate the existence of a link between node pair 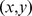. 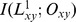 indicates the reduction in uncertainty of the connection between nodes 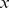 and 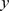 due to the information given by their common neighbors. Since the mutual information plays a significant role in our method, this framework is called *MI* for short.

If the elements of 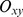 are assumed to be independent of each other, then 
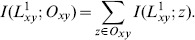
(6)


Here 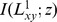 can be estimated by 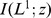, which is defined as the average mutual information over all node pairs connected to node 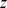


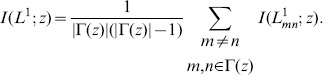
(7)


Now we try to calculate the above mutual information. According to its definition (3), 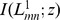 can be denoted as 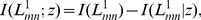
(8)where 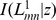 is the conditional self-information of that node pair 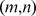 is connected when node 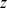 is one of their common neighbors, and 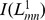 denotes the self-information of that node pair 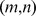 has one link. The right-hand side of eq. (8) is composed of the (conditional) self-information. Based on the definition of (conditional) self-information, it can be calculated based on the (conditional) probability.

The conditional probability 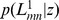 can be estimated by the clustering coefficient of node 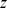, defined as 
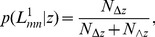
(9)where 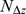 and 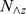 are the numbers of connected and of disconnected node pairs with node 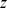 being a common neighbor, respectively. Once 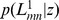 is available, 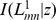 can be calculated.

In order to calculate the probability 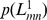, we assume that no degree-degree correlation is considered. When nodes' degrees are known, the probability that node pair 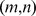 is disconnected is derived as 
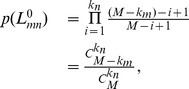
(10)where 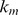 and 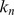 are the degrees of nodes 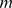 and 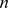, respectively. 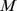 is the total number of links in the training set. Obviously this formula is symmetric, namely 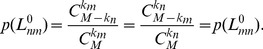
(11)


Thus, 
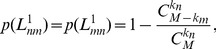
(12)and 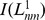 can be calculated accordingly.

Collecting these results, we can obtain 
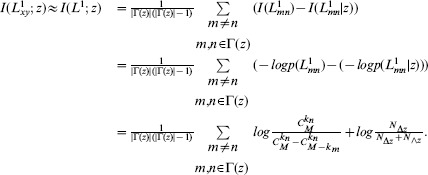
(13)It is stipulated that 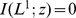 if 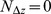.

Based on the above derivation, we have 
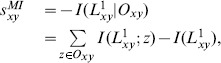
(14)where 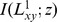 and 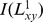 can be calculated by eqs. (13) and (12) respectively.

**Figure 1:**
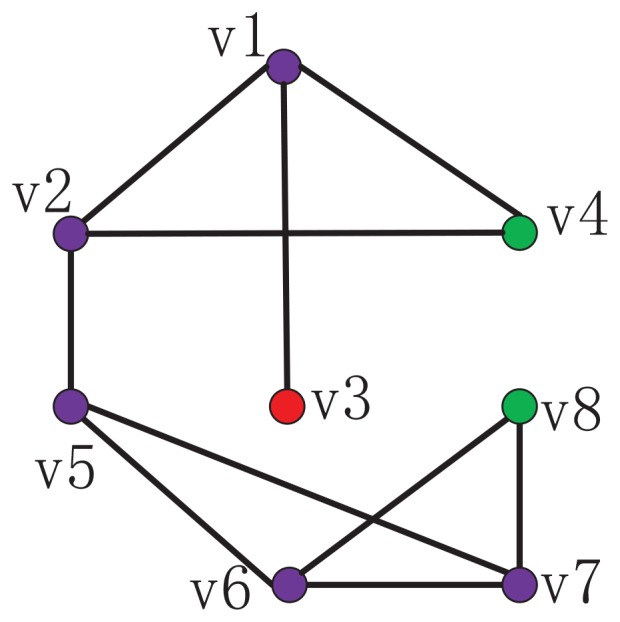
An illustration about the calculation of MI model.

To facilitate the understanding of MI, we illustrate it with an example as shown in fig. 1. First, consider node 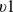, for example, which is the common neighbor of nodes 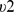, 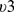 and 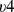. Using eq. (9), we can have 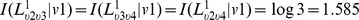. Based on eq. (12), we obtain 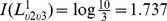, 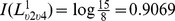 and 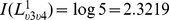. Hence, we have 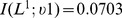. Now we compare node pairs 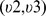 and 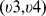 with the common neighbor node 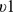. Then 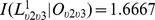, 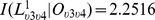, which can be calculated based on eq. (5). That is to say, node pair 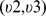 is more likely to be connected than node pair 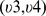. The six prediction methods mentioned in section “Previous Prediction Methods”, however, cannot distinguish these two node pairs. In this sense, MI has higher discriminative resolution than them. Second, MI can distinguish node pairs even if they all have no common neighbors. For instance, 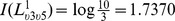 and 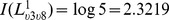. That is to say, node pair 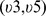 is more likely to be connected than node pair 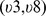. This is undoubtedly beyond the distinguishing ability of previous methods. Thirdly, the mutual information of node 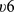 can be calculated as 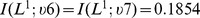. Thus 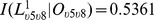. We note that 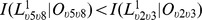, namely, node pair 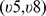 with two common neighbors has higher connection likelihood compared to node pair 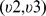 with only one common neighbor. This is in agreement with our intuition very well. Lastly, different nodes may provide different mutual information to reduce the uncertainty of connections. The extent to which node 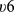 (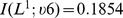) contributes to the reduction of link uncertainty, for example, is greater than that of node 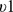 (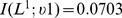).

### Experimental Results

In this section, we compare our MI approach with other six representative prediction indices which are introduced in section “Previous Prediction Methods”. Tables 1 and 2 show the prediction accuracy measured by AUC and precision, respectively. The overall prediction performance of MI outperforms them greatly.

**Table 1:** Comparison of the prediction accuracy measured by AUC on ten real-world networks.

Network 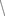 Index	CN	RA	LNB-CN	LNB-RA	CAR	CRA	MI
Email	**0.8574**	**0.8592**	**0.8588**	**0.8592**	**0.7039**	**0.7042**	0.8917
PB	**0.9233**	**0.9286**	**0.9263**	**0.9284**	**0.896**	**0.8976**	0.9322
Yeast	**0.9157**	**0.9167**	**0.9162**	**0.9165**	**0.8473**	**0.8476**	0.9368
SciMet	**0.7997**	**0.8008**	**0.8013**	**0.8013**	**0.6131**	**0.6129**	0.871
Kohonen	**0.8272**	**0.8344**	**0.8349**	**0.835**	**0.6489**	**0.6493**	0.9111
EPA	**0.6118**	**0.6131**	**0.6139**	**0.6138**	**0.508**	**0.5079**	0.9249
Grid	0.6257	0.6255	0.6258	0.6256	**0.517**	**0.5171**	0.6076
INT	**0.6523**	**0.6526**	**0.6523**	**0.6525**	**0.5277**	**0.5281**	0.9559
Wikivote	**0.9389**	**0.94**	**0.9401**	**0.9398**	**0.8899**	**0.8909**	0.9663
Lederberg	**0.9024**	**0.9058**	**0.9061**	**0.9061**	**0.7417**	**0.7414**	0.9449

Each value is averaged over 100 independent runs with random divisions of training set 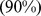 and probe set 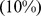. The bold font represents that MI is better than the corresponding prediction index.

**Table 2:** Comparison of the prediction accuracy measured by precision (top-100) on ten real-world networks.

Network 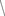 Index	CN	RA	LNB-CN	LNB-RA	CAR	CRA	MI
Email	**0.3002**	**0.2614**	**0.3236**	**0.2356**	**0.3171**	0.3442	0.3293
PB	**0.4237**	**0.2536**	**0.414**	**0.2588**	0.4795	0.4876	0.4765
Yeast	**0.6784**	**0.4989**	**0.6826**	**0.5762**	**0.6669**	**0.7664**	0.8264
SciMet	**0.1411**	**0.1265**	**0.1511**	**0.126**	0.1707	0.1791	0.166
Kohonen	**0.1577**	**0.1435**	**0.1698**	**0.1462**	**0.2097**	0.2345	0.224
EPA	**0.0156**	**0.0375**	**0.0277**	**0.0398**	**0.0271**	**0.0546**	0.0578
Grid	**0.1161**	**0.0866**	**0.1604**	**0.0968**	**0.1255**	0.1846	0.1749
INT	**0.1021**	**0.0869**	**0.1221**	**0.0636**	**0.0829**	**0.1247**	0.217
Wikivote	**0.189**	**0.1565**	**0.1875**	**0.1597**	0.2639	0.2849	0.1933
Lederberg	**0.2402**	**0.2958**	**0.2606**	**0.3075**	**0.2699**	0.3422	0.3312

Each value is averaged over 100 independent runs with random divisions of training set 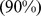 and probe set 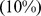. The bold font represents that MI is better than the corresponding prediction index.

Table 1 demonstrates that for AUC, MI model gives much higher prediction accuracy than all 6 other indices for real-world networks except network Grid. Especially for networks EPA and INT, AUC of six indices is all around 0.6. MI model can experience AUC of more than 0.9. Such great difference may arise from that previous methods can't distinguish those node pairs without common neighbors. Unfortunately, the lack of common neighbors between two nodes often appear in real-world networks. For example, more than 99% of node pairs in network INT have no common neighbors. But MI approach is able to discriminate them greatly. Another finding is that CAR-based indices (CAR and CRA) achieve the worst prediction performance for ten networks. Actually, for node pairs with few common neighbors, the distinguishing ability of CAR-based indices degenerates remarkably due to their emphasis on the links among common neighbors. For example, all node pairs with less than two common neighbors share the same connection likelihood because they all have no links among common neighbors.

Table 2 shows the comparisons of precision for ten real-world networks. We can see that MI is much better than CN, RA, LBN-CN, and LNB-RA for all networks. CAR-based indices, however, achieve higher precision than MI for some networks. The efficiency of CAR-based indices in predicting top-ranked candidate links is very high for networks with notable link communities. Consider, for example, network Wikivote with high average degree, in which CAR-based indices overwhelmingly win MI and other methods. Obviously, the extent to which CAR-based indices excel MI is positively related to link communities. The computing complexity of CAR-based indices, however, depends on the density of networks greatly.

It is thus necessary to compare the computing complexity of CAR-based indices and our MI model. Here the average degree is denoted as 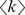. According to eq. (23), the time complexity of computing 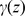 and 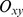 is 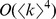 and 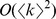, respectively. The total computing complexity of CAR is thus 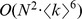. Similarly to CAR, the computing complexity of CRA is also 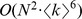 because 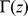 has the computing complexity of 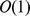 based on eq. (24). For MI, the computing complexity of 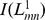 and averaging all neighboring node pairs of node 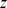 is both 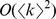. Thus, 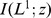 has the computing complexity of 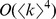. The computing complexity of MI model can be derived as 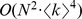 accordingly. Taking precision and the computing complexity of CAR-based indices together, we note that they outperform MI in some networks but with the computing complexity as 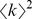 times as that of MI. It is intolerable especially for networks with the high average degree.

We also conduct experiments on an ASUS RS500-E6-PS4 workstation with 16 GB RAM and a Inter (R) Xeon (R) E5606 @ 2.13 GHz quad-core processor. The detailed comparison of computational time on ten real-world networks is summarized in Table 3. The results indicate that the MI index overwhelms CAR-based methods while remains similar time scale to other CN-based methods.

**Table 3:** Comparison of the computational efficiency of seven methods on ten real-world networks.

Network 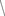 Index	CN	RA	LNB-CN	LNB-RA	CAR	CRA	MI
Email	0.115	0.201	0.161	0.161	1.65	1.64	0.472
PB	0.263	0.351	0.454	0.455	2.56	2.44	0.746
Yeast	0.414	0.802	0.499	0.499	15.3	15.3	1.97
SciMet	0.556	1.04	0.689	0.69	19.4	19.4	2.52
Kohonen	1.21	2.13	1.6	1.6	73.9	73.7	4.51
EPA	1.25	2.45	1.4	1.4	118	118	5.1
Grid	1.39	3	1.49	1.49	184	184	7.69
INT	1.75	3.56	1.9	1.91	240	239	7.69
Wikivote	5.42	8.61	9.85	9.85	573	569	19.6
Lederberg	5.38	9.8	6.9	6.9	952	952	22.3

Each value is the average time in seconds for 10 independent runs.

Altogether, MI has a good tradeoff among AUC, precision and the computing complexity.

## Discussion

In this paper, we develop a novel framework to uncover missing edges in networks via the mutual information of network topology. Note that our approach differs crucially from previous prediction methods in that it is derived from the information theory. We compare our model with six typical prediction indices on ten networks from disparate fields. The simulation results show that MI model overwhelms them. Furthermore, we compare the computing complexity of MI model with that of CAR-based indices and find that our approach is less time-consuming.

Notice that the calculation of the mutual information depends on the assumption that the network is free of assortativity. However, we find that MI method performs very well not only in uncorrelated networks but also in networks with high assortativity coefficient such as PB, Yeast and EPA. Actually, the assortativity coefficient refers to the global network-level property [33] as showed in Table 4, which can't convey sufficient property about local structure. Considering that our method is mainly focusing on the neighbors of two nodes, we utilize *local assortativity* [34], [35] to explain such a phenomenon. For a network with 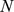 nodes and 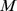 links, its excess degree (which is equal to the node's degree minus one) distribution is denoted as 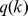. Then, the local assortativity of node 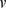 is defined as [35]

**Table 4:** The basic structural parameters of the giant components of example networks.

Network 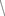 Index	N	M	e	C	r	H	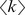	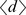
Email	1133	5451	0.2999	0.2540	0.0782	1.9421	9.6222	3.6028
PB	1222	16714	0.3982	0.3600	−0.2213	2.9707	27.3552	2.7353
Yeast	2375	11693	0.2181	0.3883	0.4539	3.4756	9.8467	5.0938
SciMet	2678	10368	0.2569	0.2026	−0.0352	2.4265	7.7431	4.1781
Kohonen	3704	12673	0.2957	0.3044	−0.1211	9.3170	6.8429	3.6693
EPA	4253	8897	0.2356	0.1360	−0.3041	6.7668	4.1839	4.4993
Grid	4941	6594	0.0629	0.1065	0.0035	1.4504	2.6691	18.9853
INT	5022	6258	0.1667	0.0329	−0.1384	5.5031	2.4922	6.4475
Wikivote	7066	100736	0.3268	0.2090	−0.0833	5.0992	28.5129	3.2471
Lederberg	8212	41430	0.2560	0.3634	−0.1001	6.1339	10.0901	4.4071

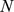
 and 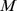 are the network size and the number of links, respectively. 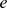 is the network efficiency [36], denoted as 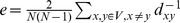, where 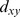 is the shortest distance between nodes 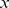 and 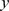. 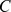 and 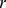 are clustering coefficient [12] and assortative coefficient [33], respectively. 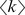 and 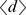 are the average degree and the average shortest distance. 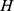 denotes the degree heterogeneity defined as 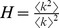.



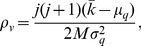
(15)where 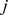 is node 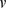
's excess degree, 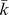 denotes the average excess degree of node 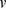
's neighbors, 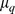 is defined as the expectation of distribution 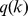 and 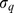 is the standard deviation of distribution 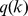. Based on this definition, the sum of all nodes' local assortativity is equal to the network assortativity coefficient. Fig. 2 shows the cumulative distribution function of nodes' local assortativity. We find that i) both locally assortative and disassortative nodes exist regardless of the network-level assortative mixing pattern; ii) most nodes do not show the local assortative property, which is coincident with our assumption. Since our method is related to the local assortativity rather than the global one, it can achieve good prediction performance even in those globally correlated networks.

**Figure 2:**
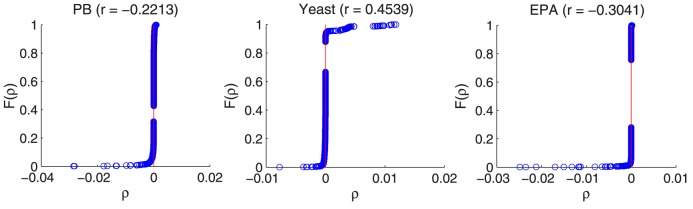
Cumulative distribution function of local assortativity, 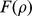 vs 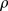, for networks PB, Yeast and EPA respectively, where 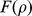 is denoted as the percent of nodes with the local assortativity value not larger than 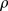. 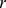
 is the assortativity coefficient of the network which is presented in Table 4.

## Materials and Methods

### Data and Problem Description

In this article, in order to better capture the statistical perspective of our method, we choose ten example data sets from various areas with the size of its giant component being greater than 1000. They are listed as follows. i) Email [37]: A network of Alex Arenas's email. ii) PB [38]: A network of the US political blogs. iii) Yeast [39]: A protein-protein interaction network. iv) SciMet [40]: A network of articles from or citing Scientometrics. v) Kohonen [40]: A network of articles with topic self-organizing maps or references to Kohonen T. vi) EPA [41]: A network of web pages linking to the website www.epa.gov. vii) Grid [12]: An electrical power grid of the western US. viii) INT [42]: The router-level topology of the Internet. ix) Wikivote [43], [44]: The network contains all the Wikipedia voting data from the inception of Wikipedia till January 2008. x) Lederberg [45]: A network of articles by and citing J. Lederberg, during the year 1945 to 2002. Here we only focus on the giant component of networks. Their basic topological parameters are summarized in Table 4.

In this paper, only an undirected simple network 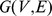 is studied, where 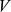 and 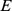 are the sets of nodes and of links, respectively. That is to say, the direction of links, self-connections and multiple links are ignored here. The framework of prediction indices can be described as follows [2]. Given a disconnected node pair 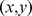, where 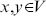, we should try to predict the likelihood of connectivity between them. For each non-existent link 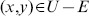, where 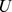 represents the universal set, a score 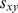 will be given to measure its existence likelihood according to a specific predictor. The higher the score is, the more possible the node pair has a candidate link. To figure out the latent links, all disconnected ones are first sorted in the descending order. The top-ranked node pairs are believed most likely to have links.

To validate the prediction performance of the algorithms, the observable links of the network are divided into two separate sets, i.e., the training set 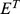 and the probe set 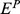. Obviously, 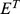 is the available topological information, and 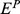 is for the test and thus cannot be used for prediction. Therefore, 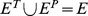 and 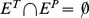. In our model, the training set 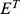 and probe set 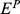 are assumed to contain 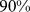 and 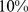 of links, respectively (see the review article [2] and references therein).

As in many previous papers, two widely used metrics are adopted to evaluate the performance of prediction algorithms [2]. They are AUC (area under the receiver operating characteristic curve) [46] and precision [47]. AUC is denoted as follows: 
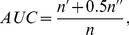
(16)where among 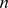 times of independent comparisons, 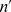 and 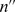 represent the time that a randomly chosen missing link has a higher score and the time that they share the same score compared with a randomly chosen nonexistent link, respectively. Clearly, AUC should be around 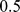 if all scores follow an independent and identical distribution. Therefore, as a macroscopic accuracy measure, the extent to which AUC exceeds 0.5 indicates the performance of a specific method compared with pure chance. Another popular measure is precision, which focuses on top-ranked latent links. It is defined as 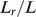, where among top-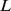 candidate links, 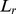 is the number of accurate predicted links in the probe set.

### Previous Prediction Methods

We here introduce six typical methods based on common neighbors. They are Common Neighbors (CN), Resource Allocation (RA) [25], the Local Naïve Bayes (LNB) forms of CN [27] and RA [27], CAR [28] and CRA [28], respectively.

- CN. This method is the natural framework in which the more nodes 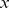 and 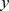 share common neighbors, the more likely they are connected. The score can be quantified by the number of their common neighbors, namely
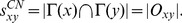
(17)
- RA. In this method, the weight of the neighboring node is negatively proportional to its degree. The score is thus denoted as
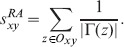
(18)
- LNB-CN. Based on the naïve Bayes classifier, this method combines CN and the clustering coefficient together. The score is defined as
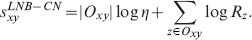
(19)

In this formula, 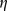 is denoted as 
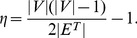
(20)

In addition, 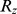 is defined as 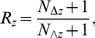
(21)where 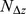 and 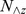 are as same as those in eq. (9)

- LNB-RA. Similarly to LNB-CN, this method takes RA and the clustering coefficient into account. The score is thus denoted as
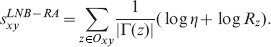
(22)
- CAR. This method boosts the discriminative resolution between latent links characterized by the same number of common neighbors through further emphasizing the link community among such common neighbors. Thus, it is described as
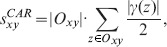
(23)where 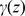 refers to the subset of neighbors of node 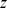 that are also common neighbors of nodes 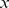 and 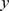.
- CRA. This method is a variation of CAR when RA is considered. It can be thus denoted as
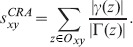
(24)

## References

[1] Liben-Nowell D, Kleinberg J (2007) The link-prediction problem for social networks. J Am Soc Inf Sci Technol 58: 1019–1031.

[2] Lü L, Zhou T (2011) Link prediction in complex networks: A survey. Physica A 390: 1150–1170.

[3] Guimerà R, Sales-Pardo M (2009) Missing and spurious interactions and the reconstruction of complex networks. Proc Natl Acad Sci USA 106: 22073–22078. 20018705 10.1073/pnas.0908366106 PMC2799723

[4] Clauset A, Moore C, Newman ME (2008) Hierarchical structure and the prediction of missing links in networks. Nature 453: 98–101. 18451861 10.1038/nature06830

[5] Yu H, Braun P, Y1ld1r1m MA, Lemmens I, Venkatesan K, et al (2008) High-quality binary protein interaction map of the yeast interactome network. Science 322: 104–110. 18719252 10.1126/science.1158684 PMC2746753

[6] Jeong H, Tombor B, Albert R, Oltvai ZN, Barabási AL (2000) The large-scale organization of metabolic networks. Nature 407: 651–654. 11034217 10.1038/35036627

[7] Zeng A, Cimini G (2012) Removing spurious interactions in complex networks. Phys Rev E 85: 036101. 10.1103/PhysRevE.85.036101 22587143

[8] Kleinberg J (2013) Analysis of large-scale social and information networks. Phil Trans R Soc A 371: 20120378. 23419847 10.1098/rsta.2012.0378

[9] Zhang QM, Lü L, Wang WQ, Zhu YX, Zhou T, et al (2013) Potential theory for directed networks. PLoS ONE 8: e55437. 23408979 10.1371/journal.pone.0055437 PMC3569429

[10] Wang WQ, Zhang QM, Zhou T (2012) Evaluating network models: A likelihood analysis. EPL 98: 28004.

[11] Barabási AL, Albert R (1999) Emergence of scaling in random networks. Science 286: 509–512. 10521342 10.1126/science.286.5439.509

[12] Watts DJ, Strogatz SH (1998) Collective dynamics of small-world networks. Nature 393: 440–442. 9623998 10.1038/30918

[13] Albert R, Barabási AL (2000) Topology of evolving networks: local events and universality. Phys Rev Lett 85: 5234. 11102229 10.1103/PhysRevLett.85.5234

[14] Kumar R, Novak J, Tomkins A (2010) Structure and evolution of online social networks. In: Link Mining: Models, Algorithms, and Applications, Springer. pp. 337–357.

[15] Sarukkai RR (2000) Link prediction and path analysis using markov chains. Comput Netw 33: 377–386.

[16] Zhu J, Hong J, Hughes JG (2002) Using markov models for web site link prediction. In: Proceedings of the Thirteenth ACM Conference on Hypertext and Hypermedia. ACM, pp. 169–170.

[17] Pavlov M, Ichise R (2007) Finding experts by link prediction in co-authorship networks. FEWS 290: 42–55.

[18] Benchettara N, Kanawati R, Rouveirol C (2010) Supervised machine learning applied to link prediction in bipartite social networks. In: Proceedings of the International Conference on Advances in Social Network Analysis and Mining. IEEE, pp. 326–330.

[19] Soundarajan S, Hopcroft JE (2012) Use of supervised learning to predict directionality of links in a network. In: Advanced Data Mining and Applications, Springer. pp. 395–406.

[20] Sá HR, Prudêncio RB (2010) Supervised learning for link prediction in weighted networks. In: III International Workshop on Web and Text Intelligence.

[21] Al Hasan M, Chaoji V, Salem S, Zaki M (2006) Link prediction using supervised learning. In: SDM06: Workshop on Link Analysis, Counter-terrorism and Security.

[22] Newman ME (2001) Clustering and preferential attachment in growing networks. Phys Rev E 64: 025102. 10.1103/PhysRevE.64.025102 11497639

[23] Leicht E, Holme P, Newman ME (2006) Vertex similarity in networks. Phys Rev E 73: 026120. 10.1103/PhysRevE.73.026120 16605411

[24] Adamic LA, Adar E (2003) Friends and neighbors on the web. Social Networks 25: 211–230.

[25] Zhou T, Lü L, Zhang YC (2009) Predicting missing links via local information. Eur Phys J B 71: 623–630.

[26] Liu H, Hu Z, Haddadi H, Tian H (2013) Hidden link prediction based on node centrality and weak ties. EPL 101: 18004.

[27] Liu Z, Zhang QM, Lü L, Zhou T (2011) Link prediction in complex networks: A local nave bayes model. EPL 96: 48007.

[28] Cannistraci CV, Alanis-Lobato G, Ravasi T (2013) From link-prediction in brain connectomes and protein interactomes to the local-community-paradigm in complex networks. Sci Rep 3: 1613. 23563395 10.1038/srep01613 PMC3619147

[29] Huang Z, Li X, Chen H (2005) Link prediction approach to collaborative filtering. In: Proceedings of the 5th ACM/IEEE-CS Joint Conference on Digital Libraries. ACM, pp. 141–142.

[30] Ahn YY, Bagrow JP, Lehmann S (2010) Link communities reveal multiscale complexity in networks. Nature 466: 761–764. 20562860 10.1038/nature09182

[31] Shannon CE (2001) A mathematical theory of communication. ACM SIGMOBILE Mobile Computing and Communications Review 5: 3–55.

[32] Cover TM, Thomas JA (2012) Elements of Information Theory. John Wiley & Sons

[33] Newman ME (2002) Assortative mixing in networks. Phys Rev Lett 89: 208701. 12443515 10.1103/PhysRevLett.89.208701

[34] Piraveenan M, Prokopenko M, Zomaya A (2008) Local assortativeness in scale-free networks. EPL 84: 28002.

[35] Piraveenan M, Prokopenko M, Zomaya AY (2010) Classifying complex networks using unbiased local assortativity. In: ALIFE. pp. 329–336.

[36] Latora V, Marchiori M (2001) Efficient behavior of small-world networks. Phys Rev Lett 87: 198701. 11690461 10.1103/PhysRevLett.87.198701

[37] Duch J, Arenas A (2005) Community detection in complex networks using extremal optimization. Phys Rev E 72: 027104. 10.1103/PhysRevE.72.027104 16196754

[38] Adamic LA, Glance N (2005) The political blogosphere and the 2004 us election: divided they blog. In: Proceedings of the 3rd International Workshop on Link discovery. ACM, pp. 36–43.

[39] Von Mering C, Krause R, Snel B, Cornell M, Oliver SG, et al (2002) Comparative assessment of large-scale data sets of protein–protein interactions. Nature 417: 399–403. 12000970 10.1038/nature750

[40] Bataglj V, Mrvar A. Pajek datasets website.

[41] Pajek datasets (nd) Available: http://vlado.fmf.uni-lj.si/pub/networks/data/mix/mixed.htm. Accessed 2014 Aug 15.

[42] Spring N, Mahajan R, Wetherall D (2002) Measuring isp topologies with rocketfuel. ACM SIGCOMM Computer Communication Review 32: 133–145.

[43] Leskovec J, Huttenlocher D, Kleinberg J (2010) Predicting positive and negative links in online social networks. In: Proceedings of the 19th International Conference on World Wide Web. ACM, pp. 641–650.

[44] Leskovec J, Huttenlocher D, Kleinberg J (2010) Signed networks in social media. In: Proceedings of the SIGCHI Conference on Human Factors in Computing Systems. ACM, pp. 1361–1370.

[45] Citation network (nd) Available: http://vlado.fmf.uni-lj.si/pub/networks/data/cite/default.htm. Accessed 2014 Aug 15.

[46] Hanely JA, McNeil BJ (1982) The meaning and use of the area under a receiver operating characteristic (roc) curve. Radiology 143: 29–36. 7063747 10.1148/radiology.143.1.7063747

[47] Herlocker JL, Konstan JA, Terveen LG, Riedl JT (2004) Evaluating collaborative filtering recommender systems. ACM Transactions on Information Systems 22: 5–53.

